# Pooling robustness in distance sampling: Avoiding bias when there is unmodelled heterogeneity

**DOI:** 10.1002/ece3.9684

**Published:** 2023-01-06

**Authors:** Eric Rexstad, Steve Buckland, Laura Marshall, David Borchers

**Affiliations:** ^1^ Center for Research into Ecological and Environmental Modelling University of St Andrews St Andrews UK

**Keywords:** abundance estimation, detectability, distance sampling, heterogeneity, pooling robustness

## Abstract

The pooling robustness property of distance sampling results in unbiased abundance estimation even when sources of variation in detection probability are not modeled. However, this property cannot be relied upon to produce unbiased subpopulation abundance estimates when using a single pooled detection function that ignores subpopulations.

We investigate by simulation the effect of differences in subpopulation detectability upon bias in subpopulation abundance estimates. We contrast subpopulation abundance estimates using a pooled detection function with estimates derived using a detection function model employing a subpopulation covariate. Using point transect survey data from a multispecies songbird study, species‐specific abundance estimates are compared using pooled detection functions with and without a small number of adjustment terms, and a detection function with species as a covariate.

With simulation, we demonstrate the bias of subpopulation abundance estimates when a pooled detection function is employed. The magnitude of the bias is positively related to the magnitude of disparity between the subpopulation detection functions. However, the abundance estimate for the entire population remains unbiased except when there is extreme heterogeneity in detection functions. Inclusion of a detection function model with a subpopulation covariate essentially removes the bias of the subpopulation abundance estimates. The analysis of the songbird point count surveys shows some bias in species‐specific abundance estimates when a pooled detection function is used.

Pooling robustness is a unique property of distance sampling, producing unbiased abundance estimates at the level of the study area even in the presence of large differences in detectability between subpopulations. In situations where subpopulation abundance estimates are required for data‐poor subpopulations and where the subpopulations can be identified, we recommend the use of subpopulation as a covariate to reduce bias induced in subpopulation abundance estimates.

## Graphical abstract

Pooling robustness is a unique property of distance sampling. This article demonstrates this property in a simulated scenario with extreme amounts of heterogeneity in detection probability between subpopulations. The article also demonstrates the bias in abundance estimates when estimating subpopulation‐specific abundance using a detection function estimated from data combined across subpopulations.

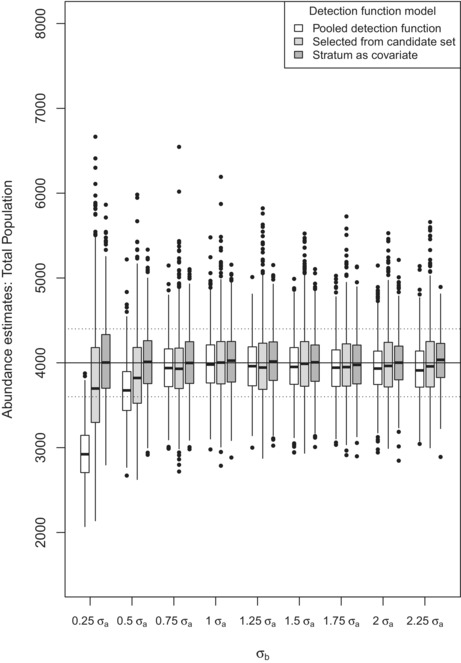

## 1 INTRODUCTION

### 1.1 Heterogeneity and pooling robustness

When estimating animal abundance, heterogeneity in the probability of capture or detection can be a major difficulty. If that heterogeneity is not adequately modeled, a large bias can ensue (Link, 2003). Although Link was specifically addressing mark‐recapture methods, he makes the following statement in the discussion: “similar difficulties are to be anticipated in the use of distance sampling models.”

Distance sampling is commonly used for the estimation of animal abundance (Thomas et al., 2010). The concept of “pooling robustness” was introduced early in the development of distance sampling (Burnham et al., 1980). It states that for standard distance sampling, unmodelled heterogeneity in the probability of detection does not generate bias in abundance estimates. Link's assertion prompted a mathematical proof that distance sampling estimators are pooling robust (Buckland et al., 2004, section 11.12).

Pooling robustness breaks down if the probability of detection on the trackline is <1. In that circumstance, it is usual to have two observers or observation platforms, and use capture‐recapture methods (mark‐recapture distance sampling, Burt et al., 2014; Laake & Borchers, 2004). As with capture‐recapture, heterogeneity must then be adequately modeled to avoid bias.

Heterogeneity in detectability can take several forms. It may be that each individual has its own detection function. In this case, in the absence of identifiable subpopulations of animals, we can rely on pooling robustness to estimate total abundance with little or no bias. Another possibility is that there are subpopulations of animals, each subpopulation with its own detection function, but we lack the information to assign detected animals to subpopulations. An example might be a population where males have different detectability from females, but individuals cannot be assigned to gender in the field. Again in this case, interest is likely to be limited to estimating total abundance as information is lacking to estimate subpopulation abundances. Thus pooling robustness again allows reliable estimation.

A third case is where subpopulations exist and individuals can be assigned to subpopulation or stratum. For example, subpopulations may correspond to different habitat types or geographic strata (Lauriano et al., 2014), or different time periods (Monks et al., 2021). In multi‐species surveys, if sample sizes are insufficient to model each species independently, each subpopulation might correspond to a species (Anderson et al., 2015). In these cases, separate abundance estimates are often needed for each subpopulation. While total abundance can still be estimated relying on pooling robustness, the separate subpopulation abundances cannot if a single detection function is fitted to the data pooled across subpopulations. It is this case that we focus on. In our experience, this issue is largely overlooked, and it is not uncommon for a single detection function model to be fitted across strata when stratum‐specific estimates are required. Examples in the literature include Allison and McLuckie (2018), Buuveibaatar et al. (2017), Edossa et al. (2022), Jefferson et al. (2021), Monks et al. (2021), and Strindberg et al. (2020).

Individuals in a subpopulation share the same detection probability. In the limit, the number of subpopulations becomes the number of individuals in the population and each subpopulation consists of but a single individual. The detection probability for the “average” individual in the population is the average of the subpopulation detection probabilities. Superimposed upon this heterogeneity in detectability between subpopulations is the change in detectability as a function of distance, with detectability being perfect at distance zero; the standard scenario for conventional distance sampling (Buckland et al., 2001). This average detection probability across subpopulations as a function of distance is a “composite” detection function.

The consequence of heterogeneity in detection probability as a function of distance is a change in the general shape of the composite detection function, compared with its components. When the amount of heterogeneity is small (detection functions differ very little between subpopulations), the shape of the composite detection function resembles the basic shape of the subpopulation detection functions. In this situation, the composite detection function can be well‐approximated by the same key function (e.g., half normal or hazard rate) as the subpopulations. Estimates of abundance based on the well‐modeled composite detection function are essentially unbiased.

**FIGURE 1:**
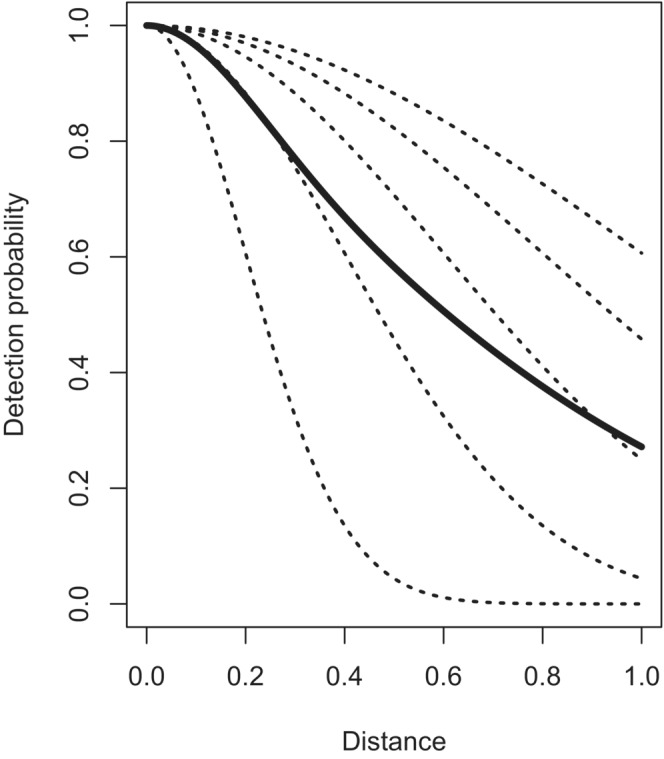
Example composite detection function (solid line) where the population is comprised of five subpopulations, each with their own detection function (dotted lines)

When heterogeneity in detectability between subpopulations is large, the shape of the resulting composite function is very different from the shapes of the subpopulation detection functions. If the composite function is modeled using the same key function as the subpopulation detection functions, the approximation will be poor. This can be seen in Figure 1 with the population equally divided into five subpopulations. Each subpopulation has a half‐normal detection function, with the scale parameter σ differing between subpopulations. Note that because of the sharp decline in detectability with distance for one of the subpopulations, the composite detection function does not mirror the shape of the detection functions of the five subpopulations, i.e., detectability drops off rapidly until the difficult‐to‐detect subpopulation becomes undetectable. Beyond this distance, the composite function falls off less rapidly. This shape is difficult to model using a half‐normal detection function model and can result in bias in abundance estimates. Fortunately, the key function plus adjustment term modeling paradigm of Buckland et al. (2001) can adequately model such composite detection functions up to a point. When the “spike” in the composite function at small distances becomes too extreme, modeling that shape becomes too difficult even for the key function plus adjustment term approach. It is in such conditions that the pooling robustness property of distance sampling is said to fail.

The alternative to modeling the composite function is to model the subpopulation detection functions. An advantage of this approach is unbiased estimates of abundance can be produced not only for the entire population but also for the subpopulations. Abundance estimates for the subpopulations based upon a model of the composite function are biased because the model of the composite function is inappropriate for any of the subpopulations (see Figure 1).

### 1.2 Modeling strategies

What is the appropriate modeling strategy when analyzing data arising from a heterogeneous population such as that depicted in Figure 1? We list four options here.

- Miller and Thomas (2015) explicitly model the heterogeneity among subpopulations rather than rely upon pooling robustness. They employ mixture models to model the average detection probability across subpopulations as a function of distance from the transect; however, the software they developed for that purpose is no longer actively maintained.
- Model the composite function using the key function plus adjustment term approach. This cannot produce unbiased estimates of abundance for each subpopulation but will produce approximately unbiased estimates of abundance for the total population. If we do not know how many subpopulations there are, and cannot assign detected animals to subpopulations, this is the only option with available software.
- Identify the subpopulations (or strata) and fit unique detection functions to each subpopulation. This should produce unbiased abundance estimates for each subpopulation, provided there are sufficient detections for each subpopulation. However, for rare subpopulations, it may be difficult to obtain sufficient detections to fit unique detection functions.
- Identify the subpopulations (or strata) and include a factor covariate corresponding to subpopulations in the detection function model. This has the advantage of producing unbiased estimates for both the subpopulation and the total population. This is the preferred option when subpopulations are known, and detected animals can be correctly assigned to them. Note that in this case, pooling robustness within subpopulations means that we can reliably estimate subpopulation abundances even when individual heterogeneity is present within each subpopulation.

This paper examines through simulation the effect of varying amounts of heterogeneity upon estimates of abundance at the population level using models of the composite detection function with (a) the key function of the subpopulations applied to the pooled data, (b) a model selected from a candidate model set including adjustment terms and an alternative key function, and (c) a covariate correctly identifying the underlying subpopulation structure. We also conduct a reanalysis of a four‐species songbird study (Buckland, 2006) where we treat the species as subpopulations.

## 2 METHODS

We investigate the effect of the degree of heterogeneity in detection probability and modeling strategy on bias in estimates of abundance. Estimation bias can only be assessed in situations where true population size is known. Varying degrees of heterogeneity in detection probability can only be induced via simulation. Therefore, our most thorough examination of heterogeneity in detection probability is performed via simulation. We also look at a real distance sampling data set to compare findings from simulations with those for a real data set.

### 2.1 Simulation study

Heterogeneity can take on many different guises. This investigation is not intended to be comprehensive in examining all forms of heterogeneity. The mimicry of heterogeneity is intentionally simplistic. We suspect the results from our investigation will generalize to other types of heterogeneity, e.g., when the number of subpopulations is equal to the number of individuals in the population, each individual possessing their own detection probability associated with a continuous covariate.

**FIGURE 2:**
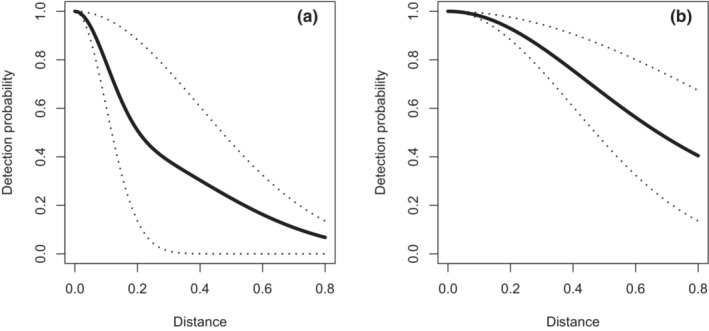
Example composite detection function (solid line) with two subpopulations (dotted lines). Panel (a) has $\sigma_{b}=\sigma_{a}/4$, while panel (b) has $\sigma_{b}=2.25\sigma_{a}$. In both panels, $\sigma_{a}=0.4$.

We explore heterogeneity in its simplest form simulating two subpopulations by dividing the study area into two geographic strata of equal size. We examine a spectrum of heterogeneity using a half‐normal key function for both subpopulations, each function determined by their respective scale parameter $\sigma_{a}$ and $\sigma_{b}$. We consider a range of heterogeneity cases, from $\sigma_{b}=\sigma_{a}/4$, through $\sigma_{b}=\sigma_{a}$ (i.e., no heterogeneity), to $\sigma_{b}\approx 2.25\sigma_{a}$. We set $\sigma_{a}=0.4$ for this series of investigations. We show the shapes of the subpopulation detection functions and of the resulting composite detection function for the two most extreme cases in Figure 2.

Detections are simulated using the dsims R package (Marshall, 2022). Estimates of abundance are produced from the resulting data using three modeling approaches and the pooled data (i.e., detections from both subpopulations): (a) model using the half‐normal key function, (b) candidate model set consisting of a half‐normal key function with 0, 1 or 2 cosine adjustment terms plus a hazard‐rate key function 0 or 1 simple polynomial adjustment terms employing AIC for model selection, and (c) a model making σ depend on a subpopulation covariate with the half‐normal key function. True abundance for each subpopulation was 2000 and truncation distance was set at $2\sigma_{a}=0.8$ for all simulations, as shown in Figure 2. One thousand replicates were conducted for each simulation scenario. Survey effort was high, with approximately 28 surveyed line transects for each subpopulation. Around 10% of each stratum occupied by a subpopulation was sampled. The number of detections for subpopulation a was approximately 120, whereas detections for subpopulation b varied as a function of $\sigma_{b}$; averaging 32 when $\sigma_{b}$ was small, up to approximately 180 when $\sigma_{b}$ was large.

Performance of the contrasting analyses methods for the estimation of total population size was measured with three metrics: percent relative bias, precision, and confidence interval coverage. To assess the ability of analytical approaches to estimate subpopulation‐specific abundance, the performance of the “true” (covariate) model was compared with the analysis employing a set of candidate models.

### 2.2 Songbird data

We revisit the investigation of Buckland (2006), specifically the point transect survey using the snapshot method, to examine various modeling strategies for a multi‐species survey. Buckland (2006) surveyed four songbird species (chaffinch (*Fringilla coelebs*), great tit (*Parus major*), robin (*Erithacus rubecula*) and wren (*Troglodytes troglodytes*)) in a 33 ha mixed woodland sampled with 32 point transect stations visited twice. In addition to the distance sampling surveys, Buckland (2006) also performed territory mapping to provide a baseline against which to compare density estimates.

Preliminary model comparison showed a preference for hazard‐rate key functions over half‐normal key functions for all single species, pooled analyses and analysis using species as a covariate. Consequently, the analyses presented focus exclusively on models using the hazard‐rate key function and treat species as the stratification criterion. We compare abundance estimates produced by detection function models in which (a) data are combined and a single detection function is fitted, (b) data are combined with a detection function that includes species as a covariate, and (c) data from each species are analyzed separately, producing a unique detection function for each species. Model (a) represents the situation in which estimates are produced at a level of aggregation below the level at which the detection function was fitted. Consequently, we expect estimates from Model (a) to violate the pooling robustness property and produce biased species‐specific density estimates.

The truncation distance was 110 m for all models. Goodness‐of‐fit was assessed for the exact distances using the Cramér–von Mises W test statistic (Burnham et al., 2004). Akaike's Information Criterion was computed for each model and we present density estimates produced for each model.

## 3 RESULTS

### 3.1 Simulation results

#### 3.1.1 Estimation of total population abundance

**FIGURE 3:**
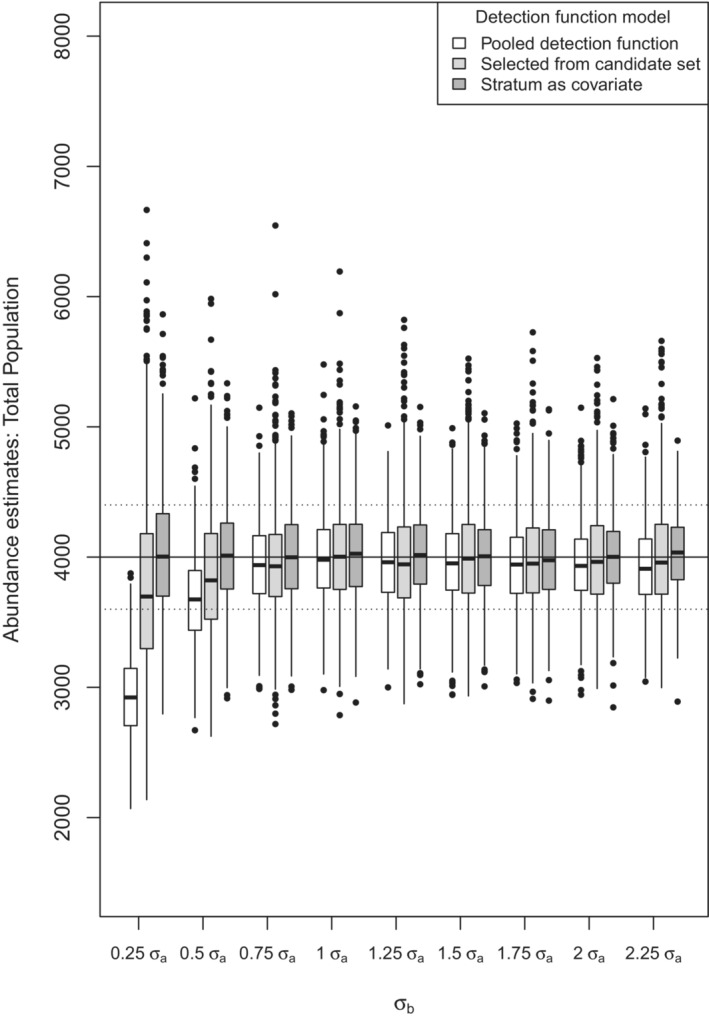
Boxplot of 1000 replicate total population estimates under differing amounts of heterogeneity in detection function (*x*‐axis) from three modeling approaches. True population size is the horizontal line across the plot. For each boxplot, the horizontal bar is a median estimate, the rectangle is the interquartile range (IQR), the vertical line is ±1.5 IQR, and circles are values more extreme than the whiskers. Percent relative bias of ±10% is shown by dotted horizontal lines.

The most distinguishing feature of the boxplots of abundance estimates (Figure 3) is the bias in the total abundance estimates for the most extreme heterogeneity case, where $\sigma_{a}=0.4$ and $\sigma_{b}=0.1$. This equates to mean detection probability in subpopulation a of $P_{a}=.60$ while detection probability in subpopulation b is $P_{b}=.16$ with a truncation distance of $2\sigma_{a}$. The bias is less extreme for models selected from a candidate set including adjustment terms, which results in more successful modeling of the composite detection function. However, the median relative bias is −27% for the key function‐only model and −8% for the candidate model set for this most extreme case. The difference in relative bias between the two modeling approaches shrinks as heterogeneity diminishes. As expected, neither modeling approach generates significant bias when the subpopulations share the same detection function scale parameter σ. Negative median bias in abundance estimates from the key function‐only models is modest for when $\sigma_{b}>\sigma_{a}$, reaching a maximum of −2.4% at the most extreme case, $\sigma_{a}=0.4$ and $\sigma_{b}=0.9$. Figure 3 shows that bias is much more extreme when the sigma multiplier is small (0.25) relative to when the multiplier is large (2.25). When the sigma multiplier is large, the resulting $\sigma_{b}$ is quite large, hence a greater proportion of detections are truncated from subpopulation b. This truncation has the effect of reducing the heterogeneity in detectability between the subpopulations.

There is more dispersion in the abundance estimates from the candidate model set compared with the key function‐only models. This is because the key function model always only estimates a single parameter, whereas the candidate model set allows for more flexibility, often estimating additional parameters in the form of adjustment term coefficient(s). The candidate model set also produces a larger number of abundance estimate “outliers,” more pronounced at extreme levels of heterogeneity, as the candidate models struggle to model the increasingly spiked shape of the composite detection function, resulting in models that predict sharp declines in detectability at small distances, consequently producing very large abundance estimates.

With respect to other performance metrics of Table 1, there is little effect of the amount of heterogeneity upon CV(total abundance estimate), except in the most extreme heterogeneity case ($\sigma_{b}=0.25,\sigma_{a}=0.1$). Confidence interval coverage is also poor in this most extreme case with the pooled detection function modeling approach. Confidence interval coverage is surprisingly good when using the subpopulation covariate in this most extreme case and remains around the nominal level for all simulated cases.

**TABLE 1:** Performance metrics for three modeling approaches in estimating total population abundance in the presence of varying levels of heterogeneity

$\sigma_{b}$	Coefficient of variation			Confidence interval coverage		
	Pooled	Candidate set	Covariate	Pooled	Candidate set	Covariate
$0.25\sigma_{a}$	0.099	0.146	0.127	0.115	0.793	0.961
$0.50\sigma_{a}$	0.090	0.106	0.097	0.840	0.883	0.955
$0.75\sigma_{a}$	0.087	0.093	0.088	0.963	0.924	0.940
$1.00\sigma_{a}$	0.084	0.089	0.084	0.945	0.911	0.956
$1.25\sigma_{a}$	0.082	0.088	0.082	0.961	0.906	0.951
$1.50\sigma_{a}$	0.081	0.087	0.081	0.944	0.907	0.949
$1.75\sigma_{a}$	0.080	0.086	0.081	0.938	0.914	0.952
$2.00\sigma_{a}$	0.079	0.087	0.080	0.947	0.922	0.947
$2.25\sigma_{a}$	0.079	0.086	0.080	0.948	0.919	0.960

#### 3.1.2 Estimation of subpopulation abundance

**FIGURE 4:**
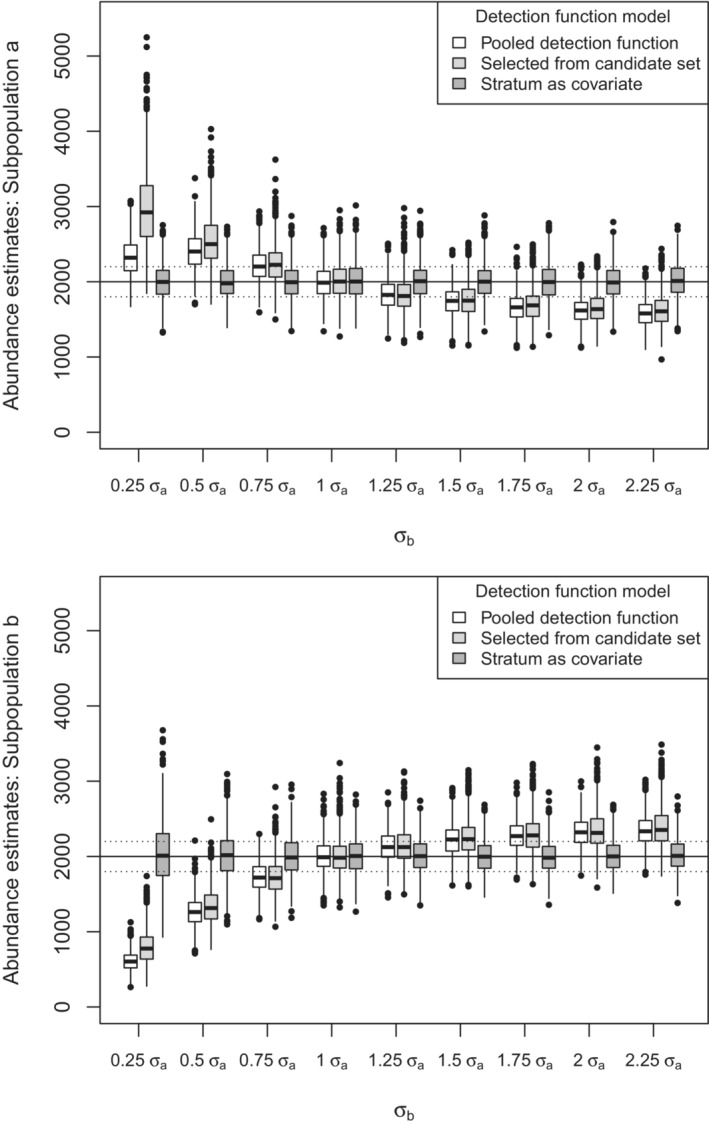
Boxplot of 1000 replicate subpopulation estimates under differing amounts of heterogeneity in detection function (*x*‐axis) from three modeling approaches. True population size is the horizontal line across the plot. Top panel contains estimates for subpopulation a, and lower panel contains estimates for subpopulation b. For each boxplot, the horizontal bar is the median estimate, the rectangle is the interquartile range (IQR), the vertical line is ±1.5 IQR, and circles are values more extreme than the whiskers. Percent relative bias of ±10% is shown by dotted horizontal lines.

As predicted in Section 1.2, abundance estimation at the level of the subpopulation is substantially biased when there is heterogeneity between subpopulation detection functions when the subpopulation identity is not recognized with the use of a covariate (Figure 4). When not modeling the detection function using a covariate, unbiased estimates of subpopulation abundance are obtained by the other modeling approaches only when the subpopulations share the same detection function (i.e., no heterogeneity) ($\sigma_{a}=\sigma_{b}$) with percent relative bias approaching ±10% when $\sigma_{b}$ differs from $\sigma_{a}$ by as little as a factor of 0.25. Abundance estimates at the subpopulation level are substantially biased. This contrasts with abundance estimation at the population level, for which the inclusion of models with adjustment terms resulted in nearly unbiased estimates of abundance (Figure 3).

Bias in estimated abundance for subpopulation b is most pronounced when $\sigma_{b}=0.25\sigma_{a}$ (Figure 2, panel a) resulting in a small number of detections and median estimated population size less than half the true population size when applying either the half‐normal key or candidate model set to fit a pooled detection function and applying that detection function to each subpopulation. Relative bias in subpopulation b abundance estimates using a pooled detection function is most profound when $\sigma_{b}$ is smallest (left side of Figure 4). This relative bias in subpopulation b abundance is smaller at the other extreme of the heterogeneity examined (right side of Figure 4) because even though the respective values of σ differ by a factor of 2.25, the detection functions decrease rather gently with distance for reasonably large values of σ represented by this scenario (see Figure 2, panel b).

There is a small decrease in abundance estimate precision as measured by CV in moving from the simplest (single parameter) pooled half‐normal model (Table 2), through the candidate model set (between one and three parameters in the model), to the model incorporating the subpopulation covariate (two parameters). Poorest precision is for the most data‐poor situation of subpopulation b when $\sigma_{b}=0.1$.

**TABLE 2:** Performance metrics for three modeling approaches in estimating subpopulation a in the presence of varying levels of heterogeneity

$\sigma_{b}$	Coefficient of variation			Confidence interval coverage		
	Pooled	Candidate set	Covariate	Pooled	Candidate set	Covariate
Subpopulation a						
$0.25\sigma_{a}$	0.107	0.152	0.119	0.712	0.269	0.948
$0.50\sigma_{a}$	0.104	0.119	0.119	0.583	0.523	0.950
$0.75\sigma_{a}$	0.105	0.110	0.119	0.841	0.846	0.949
$1.00\sigma_{a}$	0.105	0.109	0.119	0.944	0.935	0.952
$1.25\sigma_{a}$	0.105	0.110	0.118	0.867	0.811	0.937
$1.50\sigma_{a}$	0.105	0.110	0.118	0.741	0.729	0.944
$1.75\sigma_{a}$	0.104	0.110	0.118	0.580	0.625	0.924
$2.00\sigma_{a}$	0.105	0.111	0.119	0.487	0.553	0.954
$2.25\sigma_{a}$	0.105	0.111	0.119	0.404	0.474	0.960
Subpopulation b						
$0.25\sigma_{a}$	0.183	0.212	0.223	0.000	0.034	0.966
$0.50\sigma_{a}$	0.134	0.145	0.153	0.089	0.235	0.952
$0.75\sigma_{a}$	0.115	0.120	0.129	0.761	0.744	0.945
$1.00\sigma_{a}$	0.104	0.109	0.118	0.947	0.930	0.950
$1.25\sigma_{a}$	0.099	0.105	0.113	0.906	0.900	0.955
$1.50\sigma_{a}$	0.096	0.102	0.111	0.807	0.809	0.957
$1.75\sigma_{a}$	0.094	0.099	0.109	0.727	0.729	0.962
$2.00\sigma_{a}$	0.092	0.099	0.108	0.654	0.669	0.966
$2.25\sigma_{a}$	0.091	0.097	0.107	0.620	0.611	0.962

For the model including the subpopulation covariate, confidence interval coverage is at the nominal level for both subpopulations across the range of simulated heterogeneity between subpopulations. There is little to distinguish the confidence interval coverage performance of the half‐normal key function model to the candidate model set modeling. For subpopulation b, confidence interval coverage is poorest when $\sigma_{b}$ is smallest. Coverage for subpopulation b improves to nominal when $\sigma_{b}=\sigma_{a}$ and modestly declines as $\sigma_{b}>\sigma_{a}$. The pattern is similar for subpopulation a, except coverage is not so poor when $\sigma_{b}=0.25\sigma_{a}$ and the deterioration in coverage is more pronounced for subpopulation a when $\sigma_{b}>\sigma_{a}$ as a smaller proportion of detections in the sample come from subpopulation a.

### 3.2 Songbird results

**FIGURE 5:**
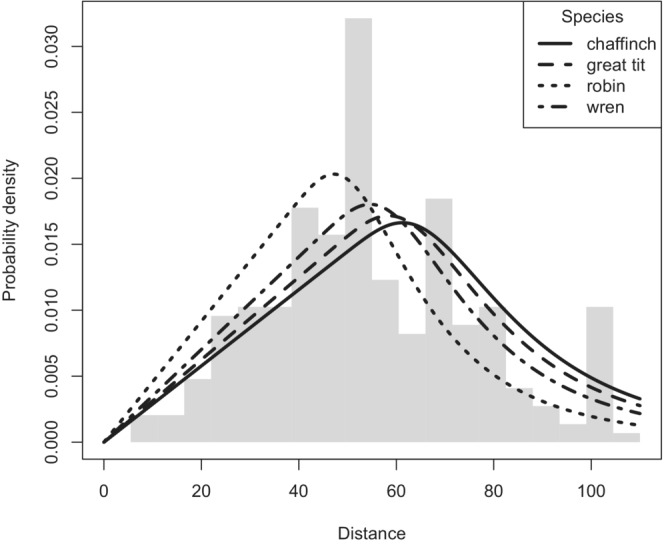
Probability density function plot of snapshot moment Montrave survey with species as a covariate in detection function. Species‐specific detection functions estimated from the population included

Data collected from Buckland (2006) for the point transect snapshot method are presented in Table 3. AIC scores for the three fitted detection function models are in Table 4. Differences in AIC among competing models are small, but the detection function using species as a covariate is the most parsimonious. Both detection function models using the pooled data possessed adequate fit to the data, assessed by the Cramér–von Mises W statistic: single detection function W=0.177,p=.317 and species‐specific detection function using species as covariate W=0.142,p=.414. Probability density functions for each species, derived from the covariate detection function model, are shown in Figure 5.

**TABLE 3:** Number of detections and species‐specific encounter rates (detections/station/visit) for Montrave snapshot point count data

Species	*n*	Encounter rate	SE (encounter rate)	CV (encounter rate)
chaffinch	81	1.266	0.127	0.100
great tit	18	0.281	0.067	0.239
robin	50	0.781	0.119	0.152
wren	117	1.828	0.157	0.086
Total	266	4.156	0.243	0.059

**TABLE 4:** AIC and ΔAIC scores for models with three modeling approaches for detection functions of Montrave snapshot point count data

Model	AIC	ΔAIC
Single pooled detection function	2387.1	1.2
Pooled data with species covariate	2385.9	0.0
Species modeled separately	2389.1	3.2

**FIGURE 6:**
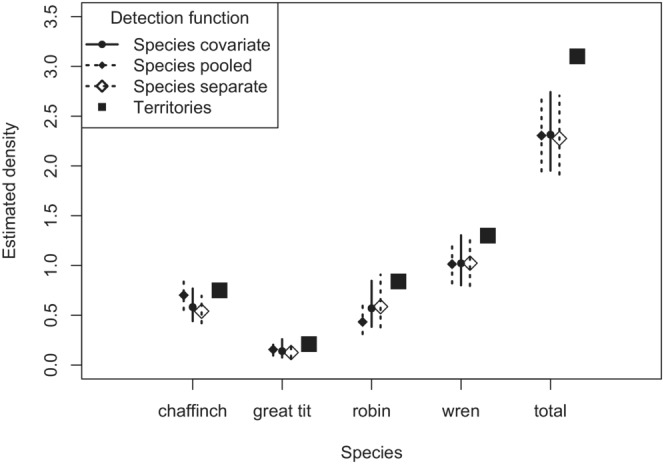
Snapshot moment species‐specific estimates with estimates computed three ways: Species as covariate, species pooled, each species with an individual detection function. Territory mapping density estimates were also provided.

Point estimates and confidence intervals for three detection function models, along with the territory mapping density estimates for each species (and summed density across species) are shown in Figure 6. The species‐specific density estimates are quite close for all competing models; the largest differences between models arise for the chaffinch and robin. Confidence intervals include the territory mapping estimates for all species when the covariate detection function model is used. Interval estimates fail to include the territory mapping estimates for the robin and wren when using the pooled detection function. Interval density estimates summed across species for all modeling approaches fail to include the territory mapping density estimate.

## 4 DISCUSSION

We have investigated the pooling robustness property of distance sampling analysis. Through simulation, we examined a range of heterogeneity in subpopulation detectability, from complete homogeneity to extreme heterogeneity. Our interest was two‐fold: to determine whether there was a level of heterogeneity that would cause the pooling robustness property to fail and induce bias in the estimation of abundance of the population, and to understand the amount of bias in subpopulation estimates of abundance caused by increasing heterogeneity when using a pooled detection function.

### 4.1 Estimation of total abundance

We succeeded in “breaking” pooling robustness and causing bias in the estimates of total population abundance. However, that breakdown (defined as |relative bias| > 10%) occurred only at extreme levels of heterogeneity, i.e., where one subpopulation possessed average detectability over distance that was 0.25 the average detectability of the other subpopulation (Figure 2 Panel a).

In reality, such extreme cases of heterogeneity could be caused by vocal male songbirds and virtually silent females attending nests. Another situation where two such different detection functions might be operating could be caused not by the characteristics of individuals in the subpopulations, but rather by the behavior of the observers conducting the survey. If one observer is searching all distances from the trackline while another observer is focusing exclusively along the trackline, radically different detection functions might apply to the two observers. In either case, differences in the histogram of perpendicular detection distances could readily be discovered through exploratory data analysis and alternative analytical strategies could be adopted, rather than relying upon pooling robustness, which would in such instances not produce unbiased estimates of population abundance. Observer training in any case should negate the possibility of such extreme heterogeneity.

The ameliorating effect upon bias in total abundance estimation from employing a candidate model set including adjustment terms is clear in Figure 3. By incorporating adjustments in the detection function model, the shape of the composite detection function can be more closely approximated, reducing bias in abundance estimation. This effect is readily seen at either end of the heterogeneity spectrum simulated in this study. The lesson is clear: include adjustment terms in a candidate model set when estimating total population abundance when heterogeneity is suspected but the source of heterogeneity cannot be identified (termed intrinsic heterogeneity by Veech et al. (2016)).

### 4.2 Estimation of subpopulation abundance

The bias‐inducing effect of heterogeneity upon subpopulation abundance estimates is much clearer than for total population abundance estimates (Figure 4). Bias in subpopulation estimates is in excess of |10%| at all levels of simulated heterogeneity when using models that did not include a subpopulation covariate. Unsurprisingly, the homogeneous instance $\sigma_{a}=\sigma_{b}$ was the only case where subpopulation abundance estimates showed no bias. The analytical lesson is clear: if the objective of the investigation is to produce estimates at the subpopulation level, explicit delineation of the subpopulation is required and included in the detection function modeling. If there is heterogeneity in detectability within subpopulations, those sources of heterogeneity need not be modeled because pooling robustness will still operate at the subpopulation level.

### 4.3 Analysis of songbird data

The effect of heterogeneity between species upon different modeling approaches using the songbird example (Figure 6) shows little departure in density estimates between the modeling approaches. This is because the heterogeneity in the detection functions between species was not great (Figure 5). Had there been a greater disparity in the species‐specific detection functions, we would expect more profound differences in density estimates between modeling approaches, with the single pooled detection function estimates exhibiting greater bias in species‐specific density estimation.

### 4.4 Generalization

Our simulations and analyses of the Montrave data contrasted abundance estimates when using either key function plus adjustment terms models or models using a covariate responsible for heterogeneity in detectability. Unsurprisingly, we found that the covariate model produced estimates with smaller bias than the model using adjustment terms.

A reviewer posed the question: how would a detection function model using covariates, but not a covariate responsible for the heterogeneity, perform relative to the key function plus adjustment terms models? There is not a universal answer to this question. Performance of a model with a “surrogate” covariate would depend upon the correlation of the surrogate with the true source of the heterogeneity. One could envision a scenario where the true source of heterogeneity is species and a surrogate of body size is used as a covariate in a survey where detections are made visually. In that situation, performance of the model using the surrogate covariate might be quite good. However, if the same surrogate covariate was used, but detections were made aurally (such that there is little correlation between body size and detectability), then the performance of the surrogate covariate model might be poorer than the key function plus adjustment term detection function model.

Producing unbiased abundance estimates for subpopulations is best accomplished when there are adequate data to estimate a detection function for each subpopulation. If adequate data are not available for all subpopulations, then modeling the detection function using subpopulation as a covariate will still produce unbiased abundance estimates. Employing covariates in the detection model that are proxies for subpopulation may produce adequate results, but that is dependent upon the quality of the proxies to identify subpopulations. The use of adjustment terms when the source of heterogeneity is unknown does not remove bias from estimates of subpopulation abundances.

## 5 CONCLUSION

**FIGURE 7:**
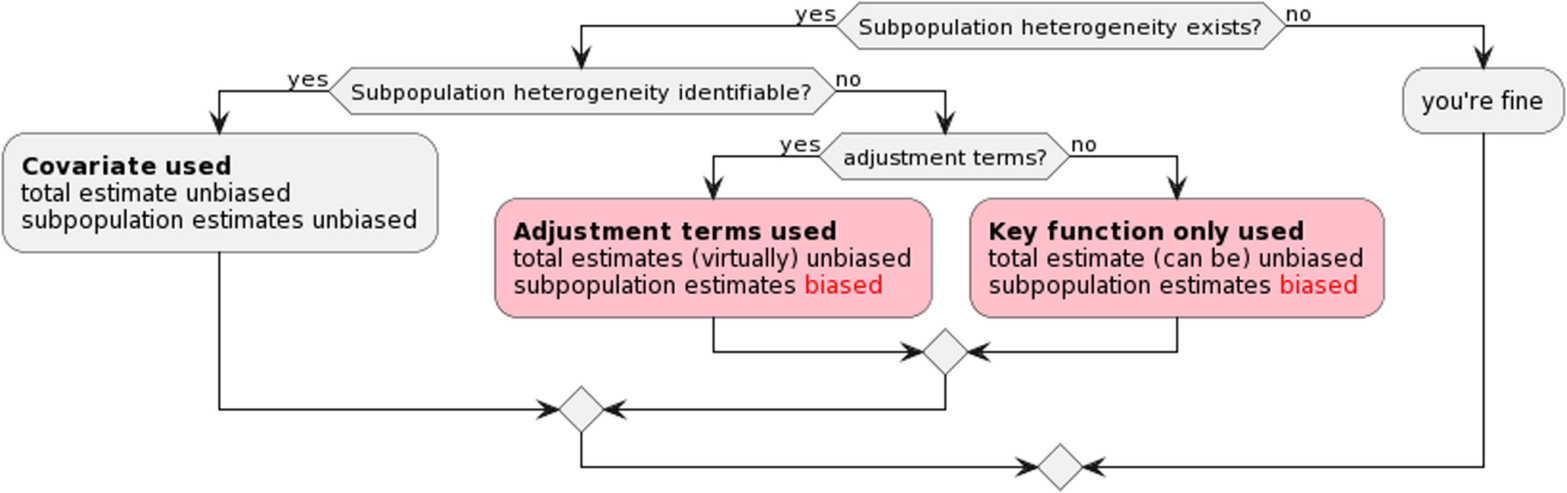
Analytical scenarios that include the presence of subpopulation heterogeneity in the detection function along with the estimation of abundance at the population or subpopulation level. Likely outcomes of modeling strategies described in this paper are highlighted.

There are countless factors that may induce heterogeneity into the detection process in distance sampling surveys. Modeling resulting heterogeneity through the use of covariates can be complex, as it is in capture‐recapture analyses. However, not all sources of variation in detectability need to be modeled to generate reliable estimates, thanks to the pooling robustness property of distance sampling. Unbiased estimates of population abundance can be obtained courtesy of the pooling robustness property. However, if estimates at the subpopulation level are desired, then the inclusion of a subpopulation covariate will produce unbiased abundance estimates at the subpopulation level, even when some subpopulations have a small number of detections (Figure 7).

A candidate model set that includes models with key functions and adjustment terms plus a model with the subpopulation covariate can be adopted, and subjected to information‐theoretic comparison to assess the utility of the subpopulation covariate. Estimation under the subpopulation covariate model may be preferred in any event, unless so many subpopulations are identified resulting in a large number of parameters in the covariate detection function model. In some circumstances, this may lead to unacceptable erosion in the precision of the abundance estimates.

The use of software that permits the fitting of such models along with a model selection procedure to ensure an adequate fit to the data (e.g., Miller et al., 2019; Thomas et al., 2010) is central to using the pooling robustness property of distance sampling to the investigator's advantage. This approach should be employed whether the objective of the survey is to obtain unbiased estimates of subpopulation or total population abundance.

## AUTHOR CONTRIBUTIONS

**Eric Rexstad:** Conceptualization (lead); formal analysis (lead); methodology (equal); writing – original draft (lead); writing – review and editing (equal). **Steve Buckland:** Investigation (equal); methodology (equal); writing – original draft (equal); writing – review and editing (equal). **Laura Marshall:** Software (supporting); writing – review and editing (equal). **David Borchers:** Investigation (equal); writing – review and editing (equal).

## ACKNOWLEDGEMENTS

The authors acknowledge helpful comments of two anonymous reviewers as well as curiousity of participants in training workshops that prompted this investigation of pooling robustness.

## CONFLICT OF INTEREST

The authors express no conflicts of interest.

## Data Availability

Data analyzed in this manuscript are from a previously published paper by Buckland (2006). The csv file for point transect survey employing the snapshot method for the four songbirds can be accessed via https://doi.org/10.5061/dryad.k98sf7m95.
